# Episodic Memory, Chiari I Malformation, Personality and Coping: The Role of Chronic Pain

**DOI:** 10.3390/bs15121678

**Published:** 2025-12-04

**Authors:** Philip A. Allen, Kevin P. Kaut, James R. Houston, Michelle L. Houston, Emily P. Rabinowitz, Douglas L. Delahanty, Petra M. Klinge

**Affiliations:** 1 Department of Psychology, The University of Akron, Akron, OH 44325-4301, USA; 2 Department of Psychology, Middle Tennessee State University, Murfreesboro, TN 37132, USA; 3 Department of Surgery, Vanderbilt University Medical Center, Nashville, TN 37232, USA; 4 Department of Psychological Sciences, Kent State University, Kent, OH 44240, USA; 5 Department of Neurosurgery, Rhode Island Hospital, and Warren Alpert Medical School, Brown University, Providence, RI 02903, USA; petra_klinge@brown.edu

**Keywords:** episodic memory, chronic pain, Chiari malformation, allostatic load, drug effects on episodic memory

## Abstract

Episodic memory is contextual memory linking temporal, spatial, emotional, and self-referential contexts. In this review, we placed particular emphasis on the emotional context because of its nexus with chronic pain effects. Psychological (e.g., depression, anxiety, stress, & loneliness) and medical (neurological, cardiovascular, chronic pain) conditions can adversely affect episodic memory. Furthermore, individual differences in emotional/affective experience as represented by trait personality variables (e.g., conscientiousness, openness to experience, introversion, extraversion, and neuroticism) can either facilitate or harm episodic memory performance. This paper aims to review episodic memory, its associated attention and executive function co-processes, the modulation of memory function as a result of affective experiences as represented by trait personality, and how coping mechanisms can serve as a buffer to maintain episodic memory function using Chiari malformation Type I (a chronic pain syndrome) as a model. Finally, allostatic load and pharmacological effects of pain medications on episodic memory are discussed.

## 1. Background/Aims

Episodic memory is linked to chronic pain syndromes. In the present paper, we emphasize how emotional/affective contextual coding and individual differences in affective experience as represented by trait personality characteristics relate to healthy coping mechanisms in healthy individuals as well as applications of episodic memory issues in medical syndromes. Emotional coding in episodic memory is typically more central to medical conditions associated with chronic pain (e.g., low back pain, diabetic neuropathy, arthritis, fibromyalgia, and Chiari malformation Type I, or CM1) than are temporal, spatial, and self-referential coding contexts. The present review of episodic memory begins by discussing the psychological and neuroscience approaches to episodic memory. Next, we discuss how working memory and attention interact with episodic long-term memory, noting that focused attention can lessen the negative effect of pain on episodic memory. The subsequent section describes the importance of emotional context in episodic memory with particular emphasis on its links to the chronic pain syndrome of Chiari malformation Type I (or CMI). This section also discusses nociceptive (sensory) and centrally sensitized (sometimes referred to as neuropathic) pain types and how individual differences in trait personality characteristics and psychotherapy interventions (acceptance and commitment therapy, or ACT) are related to episodic memory. A critical study is then discussed that provided evidence that individual differences in self-focused attention, or reflection (Trapnell & Campbell, 1999), helped CMI patients suppress pain and improve episodic memory performance. We then complete this review by discussing how allostatic load (Juster et al., 2010; McEwen & Stellar, 1993) and pharmacological therapies can affect (negatively or positively) episodic memory performance.

## 2. Introduction to Episodic Memory and Its Psychological Mechanisms

It is important to make a distinction between conscious and nonconscious memory effects. Explicit memory (conscious memory) and implicit memory (memories of which one is not consciously aware) have typically been considered separate types of episodic memory (Squire, 2004). However, more recent evidence suggests that explicit and implicit memory may have common neural mechanisms (Dew & Cabeza, 2011). For example, the traditional conceptualization of intense emotional flashbulb memories placed emphasis on their implicit encoding nature. Yet, more recent work suggests that perhaps the implicit flashbulb memories and explicit memories do share brain processing (e.g., Dew & Cabeza, 2011). For example, Brown and Kulik (1977) provided evidence that flashbulb memories often result in accurate retrieval even though encoding is automatic. If this is the case, then this provides additional evidence of the efficacy of emotional context in explicit episodic memory. However, we will emphasize explicit memories in the present review.

Episodic memory involves the conscious recollection of an event and the context in which the event occurred (Tulving, 1983), including temporal, spatial, emotional, and self-referential contexts (Allen et al., 2008). Episodic memory also includes “autonoetic awareness/consciousness” (Tulving, 1985), referring to the ability to think about oneself. Applied to episodic memory, autonoetic awareness is the ability to remember events that occurred to you, as well as a time perspective of things that will happen to you in the future (prospective memory). Alternatively, semantic memory reflects our general knowledge of the world and involves noetic awareness—which allows an individual to be aware of concepts and relations among concepts that are not present (Tulving, 1985). For example, a previously prepared mental list of specific items needed for purchase at the local hardware store would involve episodic memory. However, if we could not remember the items on this mental list, we might use our general knowledge of common things that one might purchase at a hardware store (e.g., semantic memory). A third type of memory (in addition to episodic and semantic) is autobiographical memory. This differs from episodic memory by involving both memories about oneself’s episodic memory with the self as a context and semantic knowledge of oneself (Marsh & Roediger, 2013).

Most models of episodic memory include two or three stages. Two-stage models include an input or encoding stage that involves short-term memory/working memory (STM) thought to be associated with prefrontal brain function (D’Esposito & Postle, 2002) and a storage stage that involves long-term memory (LTM). This LTM stage involves associating a STM event with a context, a phenomenon termed hippocampal indexing (Aly & Turk-Browne, 2016; Teyler & Discenna, 1986). Neuroscience-based models typically include the first two stages in addition to a process of information consolidation (Allen et al., 2019), assumed to result in long-term storage involving protein synthesis and neural plasticity requiring hours to days (McGaugh, 2000). Ironically, most human LTM research paradigms involve a relatively short retention interval—frequently 30 s, or less—as illustrated by the Brown-Peterson distractor task methodology (Peterson & Peterson, 1959). For example, Glanzer (1972) showed that when no distractor task was used in a serial recall task, there was a robust recency effect (suggesting that the last several items in the list remained in short-term memory). However, when the same serial recall task also included a distractor task, the recency effect was eliminated (suggesting that these last few items needed to be retrieved from long-term memory) (Glanzer, 1972), whereas the non-human animal LTM research literature emphasizes consolidative mechanisms and reductionist processes facilitating the transition of short-term physiological correlates of experience into long-term neural modifications embedded within cortical cytoarchitecture (McGaugh, 2000); this is not typically considered in human episodic memory research—although it has been explored in the work of (Allen et al., 2019).

## 3. Episodic Memory: Emotional/Affective Context

Spatial, temporal, and self-referential contexts in episodic memory have received considerable research and theoretical attention in the episodic memory literature. However, there is an increasing acknowledgement that more research and theoretical integration are needed to understand affective contextual coding in episodic memory (Allen et al., 2008). Indeed, coding for affective context in episodic memory has been shown to be especially effective for the success of episodic memory retrieval (Kensinger, 2009; LaBar & Phelps, 1998) as well as the feeling of remembering (Sharot et al., 2004). An early example of emotional context influencing episodic memory was Gordon Bower’s research on mood congruence (Bower, 1981), which showed that memories are easier to recall when the emotional state at retrieval matches that at encoding. Further extending our understanding of this connection between emotional state and memory, Damasio’s somatic marker hypothesis underscores how emotion linked bodily responses (e.g., bodily/somatic senses; alterations in physiology and physical activity—elevated heart rate) become integrated with episodes of experience. The neural encoding of an episode and attendant emotional context yield ‘markers’ of previously experienced events, thus tagging or indexing these multidimensional experiences. Clinically, evidence from patients with ventromedial prefrontal cortex damage (Bechara et al., 2005; Damasio, 1994) (a classic example of ventromedial prefrontal damage is the case study of Phineas Gage) has reinforced a potential neural model for the influence of emotion on episodic memory (Allen et al., 2005).

## 4. A Neuroscience Model of Episodic Memory

The human and non-human animal literature suggest that the hippocampus (which receives diverse pre-processed sensory inputs from multi-modal sensory cortices) (Squire & Zolamorgan, 1991) is a critical component in a system associated with the development of context-dependent and event-specific representations by integrating or otherwise “indexing” distributed patterns of neocortical activity driven by sensory experience (Teyler & Discenna, 1986). Damage to the hippocampus in humans has been shown to undermine the formation of event-related memories experienced both before and after damage (i.e., retrograde and anterograde amnesia, (Calabrese et al., 1996; Corkin, 1984; Kartsounis et al., 1995; Reed & Squire, 1997, 1998; RempelClower et al., 1996), and interferes with the acquisition of spatial-relational information (Teng & Squire, 1999).

In addition to the hippocampus, other brain areas associated with the episodic system include the thalamus (i.e., facilitating circuit connectivity), mamillary bodies (posterior region of the hypothalamus), amygdala (anterior to the hippocampus in the medial temporal lobes), and the prefrontal cortex (Allen et al., 2008). Interestingly, the often-reported experience of emotional content embedded within an episodic recollection is posited to reflect a highly conserved neural system designed to integrate emotions with stimulus events occurring as part of a specific event. In particular, the aforementioned amygdala and prefrontal cortex are held to be essential processing regions for integration of emotional experience related to episodic events. For example, the role of the amygdala in negative emotions such as fear and anxiety is well known (LaBar & Cabeza, 2006; Leigland et al., 2004), and the ventromedial prefrontal cortex is implicated strongly in regulating emotional behaviors (Damasio, 1994). Brain imaging studies reveal patterns of neural output from the amygdala to the prefrontal cortex during emotional processing, thus underscoring an amygdalar-prefrontal contribution to episodic memory modulation (Kilpatrick & Cahill, 2003) and supporting a potential mechanism whereby emotionally coded stimuli might strengthen/consolidate certain memories (Ikeda et al., 1998). Also, frontal-mediated processes (Cabeza, 2001, 2002; Langley & Madden, 2000) and amygdala-related affective modulation (LaBar & Cabeza, 2006) serve to integrate spatial, temporal, and affective coding, the multiple sensory events that occur as components of a specific episode (Nadel & Moscovitch, 1998).

This suggests that the neural system driving episodic memory converges with the emotional regulation system, thereby aligning these processes to a much greater extent than the somewhat separate, albeit related, semantic memory network (Menon et al., 2002). This anatomical convergence, recognized by Papez as early as 1937, continues to be characterized and understood as a modulator of affective and sensory experiences involved in emotional regulation and memory development (Kamali et al., 2023). Central to the present review addressing the research regarding Chiari Malformation Type I, chronic pain, and episodic memory, it is instructive to note that the cerebellum, via connectivity with the hypothalamus and basal forebrain regions, is likely to support autonomic, cognitive, and affective processes (Kamali et al., 2018). The anatomical connection between the cerebellum and distributed networks influencing diverse aspects of motor, cognitive, and affective behavior permits further reflection on how conditions impacting cerebellar afferent and efferent pathways (e.g., Chiari Malformation) might explain even subtle evidence of episodic memory and emotional processing concerns. It would be of further importance to identify clinical examples involving the relevant anatomy discussed here that might naturally allow us to discern the relationships among emotion, attention, episodic and semantic memory.

Despite similar connectivity among episodic and semantic memory systems, current evidence suggests that semantic knowledge in humans is less dependent on hippocampal-frontal cortex connections, and more specifically associated with a broader neural network including the medial temporal cortex (Allen et al., 2008; Davies et al., 2004; Hodges & Patterson, 1997; VarghaKhadem et al., 1997), the inferior and lateral temporal cortex (Kapur et al., 1994), and related cortical regions (Demonet et al., 1992; Petersen et al., 1988; Posner et al., 1988). Neuropsychologically, it would make sense—given our current understanding—that cerebellar impingement or alteration, as in Chiari Malformation (Type I), would impact cognitive processes, the extent of which might be greatest at the intersection of emotional reactivity and episodic memory expression. Explanatory mechanisms require further exploration and elucidation, but we attempt here to offer an integrative perspective guiding ongoing theoretical and empirical work concerning the neurocognitive expression of memory and emotion.

## 5. Damasio’s Somatic Marker Hypothesis

**Figure 1:**
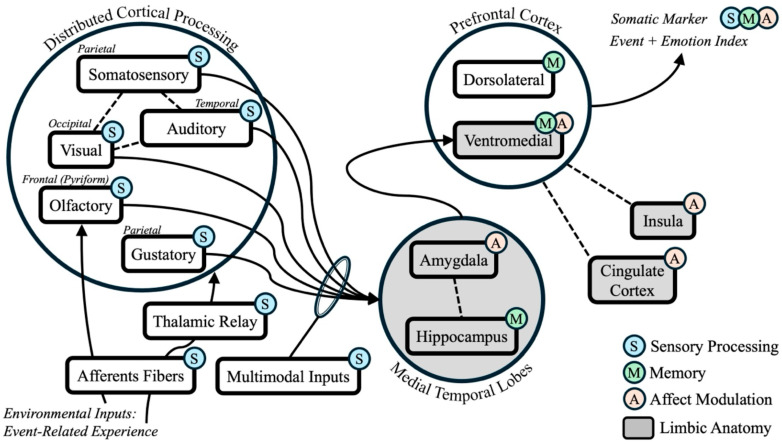
Conceptual model for somatic marker formation in episodic memory. Note. The solid arrow represents the cascade of signaling hypothesized to result in somatic markers. The dashed line represents relevant interconnectivity of structures.

Damasio’s somatic marker hypothesis is principally concerned with how emotional context plays a crucial role in decision-making through visceral cueing (Bechara et al., 2005; Damasio, 1994). This framework supports the notion that emotional context results in somatic (i.e., physical/sensory experiences), or affect-driven contextual markers facilitating (or possibly undermining) episodic memory depending on the circumstances (refer to Figure 1 for the contextual marker perspective in episodic memory) (Allen et al., 2005, 2008). Allen and colleagues previously offered support for the role of somatic markers in episodic memory by identifying a mediating effect of trait neuroticism on memory performance in healthy younger and older adults (Allen et al., 2005). Older adults showed significantly lower trait neuroticism scores than their younger adult counterparts. Additionally, older adult episodic memory performance was poorer than younger adults’ performance. Critically, this inverse relationship between age and memory performance was mediated by trait neuroticism. That is, neuroticism level significantly accounted for the correlation between age and episodic memory. This finding, combined with evidence that aging is associated with a reduced neurophysiological response to affective stimuli (Levenson et al., 1991, 1994), suggests that older adults place less vivid affective contextual markers on to-be-remembered stimuli than do younger adults.

**Figure 2:**
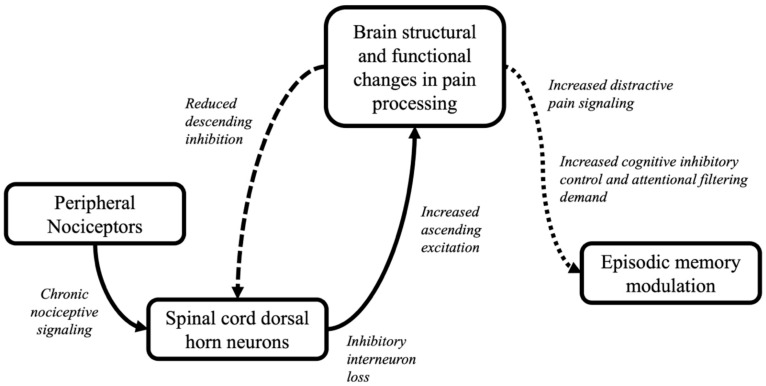
Conceptual model of the central sensitization of pain and resulting modulation of episodic memory function. Note. The solid arrows represent ascending nociceptive paths. The dashed line represents descending inhibition of pain that is disrupted when central sensitization occurs. The dotted line reflects the functional impact of central sensitization on episodic memory function.

As developed in Figure 1, Allen and colleagues’ perspective of emotion-mediated memory incorporates a neurocognitive framework established by the somatic marker hypothesis, thereby emphasizing the emotion processing role of the ventromedial prefrontal cortex (VMPFC) in memory formation. Evidence regarding the limbic system (e.g., see shaded areas in Figure 2) supports the contributions of individual structures and the collective network in forming integrated and organized representations of experiences in ‘neural space’ (Fabri et al., 2021). In particular, accumulating evidence suggests that individuals with VMPFC damage exhibit an episodic memory deficit (Bertossi et al., 2016; McCormick et al., 2018; Melo et al., 1999). In particular, evidence suggests that the VMPFC is central to the scene construction process in episodic memory (McCormick et al., 2018), Most importantly, the VMPFC forms a core component of the episodic memory system in conjunction with the widespread cortical processing of sensory inputs converging on the hippocampus via the entorhinal cortex, filtered also—as necessary—by the amygdala, notable for its role in negative affect and attending to probable threats (Alexandra Kredlow et al., 2022). The organization of this system is consistent with Damasio’s framework, and as extended here to include affective processing regions such as the cingulate cortex and insular region notably aligns with the conceptualization of negative affective as a contextual marker, including negative emotionality associated with trait neuroticism levels (Allen et al., 2005). Note that the VMPFC is also a hub of the broader default mode brain network (DMN), which is also associated with episodic memory function as well as emotional processing (Menon, 2023; Shapira-Lichter et al., 2013).

## 6. Working Memory and Attention Modulation of Episodic Long-Term Memory

A key assumption in the episodic memory construct is that the central executive process subserving working memory capacity (i.e., memory span) is limited, which naturally affects the organization and status of both short term and long-term memory. New information is constantly entering STM through the form of coherent neural transmissions, or “codes”, that are transiently maintained. To maintain, or “encode,” STM codes into permanent storage units, these must be converted to an LTM code that no longer depends on conscious awareness (Tulving, 1983). Otherwise, such codes would be lost to displacement or interference. Essentially, when a more permanent LTM code is brought back into conscious awareness, this requires a retrieval process to actively search through an ostensibly limitless and dynamic memory architecture. Importantly, memory encoding and retrieval—each considered a cognitive control process—involve selective attention and working memory.

A central tenet of the current review is that among the various factors that can easily distract from, or interfere with episodic memory, the physical and emotional experience of pain is a stimulus event that can undermine memory development. In particular, chronic pain can be viewed as a prepotent (i.e., over-riding) distractor creating interference by occupying limited attentional capacity, thereby negatively impacting episodic memory performance (Eccleston & Crombez, 1999). Accordingly, an important coping mechanism for chronic pain patients is developing strategies or using personality characteristics to inhibit pain.

## 7. Chronic Pain as a Modulator of Episodic Memory and the Link to Chiari Malformation

Pain is known to interfere with cognitive processes such as episodic memory (Eccleston & Crombez, 1999). Classical pain research has primarily focused on nociceptive, or sensory, pain (Melzack & Wall, 1965), which arises from physical stimuli that cause tissue damage (e.g., pressure, temperature, or corrosive material) and activates a pain receptor that transmits a pain signal to the brain (Melzack & Wall, 1965). While the classic gate control theory of nociceptive pain (Melzack & Wall, 1965) has been shown to have some limitations (e.g., explaining phantom limb pain), the core principles still largely hold (Mendell, 2014). However, to adequately explain chronic pain syndromes, such as phantom limb pain, diabetic neuropathy, lower back pain, and rheumatoid arthritis and CMI, the introduction of central sensitization of pain was necessary. Centrally sensitized pain refers to pain responses in the brain independent of a sensory signal (Al Samman et al., 2024; Allen et al., 2018, 2024; M. Garcia et al., 2022). Note that centrally sensitized pain begins as acute nociceptive pain—but when these nociceptive signals become chronic, the brain begins to develop chronic pain in the absence of a nociceptive sensory signal (see Figure 2 for an illustration of nociceptive versus centrally sensitized pain). This concept appears to be analogous to the phantom limb phenomenon, in that the brain has decided that one is “in pain” even without a nociceptive (sensory) pain signal in the same way that a person who has lost a limb still “feels” the limb. This process is also sometimes referred to as neuropathic pain, commonly measured using self-report tools such as the McGill pain questionnaire series (Dworkin et al., 2009).

**Figure 3:**
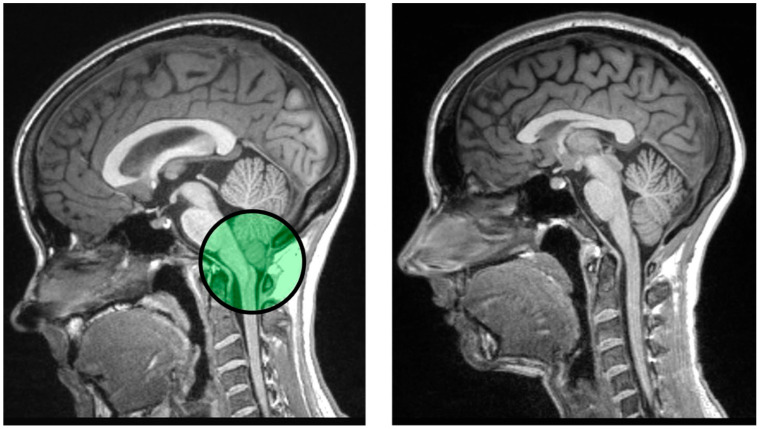
MRI presentation of Chiari Malformation Type I and a healthy control. Note. T1-weighted structural midsagittal images are provided. The CMI patient image is presented to the left. A healthy control is presented to the right for reference. The green area highlights the displacement of the cerebellar tonsils below the foramen magnum. This displacement acts as the pathognomonic radiological feature of CMI.

While many disease models can be successfully applied to the study of neuropathic pain, we reference Chiari malformation type I (CMI) as a case example. CMI, is radiologically characterized by cerebellar tonsils displaced 5 mm or more below the foramen magnum (i.e., the anatomical feature through which the brainstem descends and transitions into the cervical spinal cord), see Figure 3 (Eppelheimer et al., 2018; Friedlander, 2024; J. R. Houston et al., 2018; Leonard & Limbrick, 2015; Milhorat et al., 1999). The cerebellar tonsillar descension appears to be precipitated by a combination of cervico-medullary compression combined with the cardiac cycle (i.e., heartbeat) and respiration (breathing) resulting in pressure waves acting as a “water hammer” (Clarke et al., 2013a, 2013b; Doberstein et al., 2017; Friedlander, 2024; Heiss, 2023; Ibrahimy et al., 2023; Leung et al., 2016; Milhorat et al., 1999). Over time, this increased CSF pressure can result in lower dural compliance at the craniocervical juncture in CMI patients (Labuda et al., 2022b) due to the stiffening of the dura mater and strain on the myodural bridges (Klinge et al., 2021; McElroy et al., 2019) in the cervical spine. Also, CMI patients have been shown to have smaller anterior CSF space between the 2nd and 4th cervical vertebrae (Allen et al., 2024; M. Garcia et al., 2022) which likely compresses pain pathways between the nerve roots of C2 and C4 that project to the posterior dura of the brain (Noseda et al., 2019). Critical to the present application, the posterior dura is the location in which occipital headaches predominate (Doberstein et al., 2017). These fluid-mechanical compliance and compression phenomena can result in a diverse CMI symptom profile affecting motor, cognitive, clinical and sensory systems, including a neurological pain syndrome (Fischbein et al., 2015; M. Garcia et al., 2024; M. A. Garcia et al., 2019; Tokar et al., 2023). While CMI occurs for children and adults, the presentation is somewhat different in adulthood than in childhood. In childhood, girls and boys appear to be equally affected, whereas approximately 80% of adult CMI patients are females (Allen et al., 2024; Wilkinson et al., 2017). As defined by Chicago Chiari Outcome Scale (or CCOS) scores (Aliaga et al., 2012; Yarbrough et al., 2014), Allen and colleagues observed that women show lower scores (indicating a poorer surgical outcome) than did children (girls or boys) and men—and that this effect was associated with smaller anterior CSF space in women than in the other three groups (Allen et al., 2024). Finally, and critical to the present topic of episodic memory there is evidence that CMI patients show episodic memory dysfunction—and that this is related to pain (Allen et al., 2018)—this will be discussed in more detail in a later section.

Chronic pain, particularly in the head and neck area, as is typical in CMI, and various indicators of pain symptomatology have also been shown repeatedly to correlate with cognitive performance and underlying neuroanatomy and physiology (J. R. Houston et al., 2020, 2022; M. L. Houston et al., 2021). Curiously, despite experiencing long-term chronic pain, self-report research has not shown significant correlations between neuropathic pain index scores and pain-related symptoms in CMI patients (Tokar et al., 2023). However, the pain-related symptoms of the same sample of CMI patients were correlated with another classification of pain—namely, affective pain. Affective pain refers to the emotional experience of pain that can be modulated by mood states. As such, affective pain is an ideal target for intervention to help those living with chronic pain like CMI patients (e.g., by teaching better coping skills). Affective pain in CMI also appears to be linked to central sensitization of pain in which the brain decides that the body is in pain—even without the presence of sensory (nociceptive) pain (Allen et al., 2018, 2024). Thus, while clearly connected, nociceptive, neuropathic, and affective pain are uniquely associated with pain experience. Because prepotent chronic pain distracts the central executive in working memory from the focus on encoding and retrieval, the present review places particular emphasis on the pathogenesis of central pain sensitization and coping mechanisms that allow individuals with chronic pain to optimize their episodic memory performances.

## 8. Theoretical Application: Pathogenesis of Central Pain Sensitization in Chiari Malformation Type I

It is helpful to consider what makes nociceptive pain convert to centrally sensitized pain. As noted above, this conversion appears to require a sustained nociceptive signal, leading to the development of centrally sensitized signals occurring within the diverse network for pain processing. CMI may again serve as an informative case example. In CMI, accumulating evidence suggests that cerebrospinal fluid compression (i.e., increased pressure) within the anterior cervical spine area between the foramen magnum and the C2 vertebrae are associated with increased pain (Allen et al., 2024; M. Garcia et al., 2022; Gholampour & Gholampour, 2020; Gholampour & Taher, 2018). As noted earlier, this compression could result from a loss of cranio-cervical compliance (Goel, 2015; Goel et al., 2020; Klinge et al., 2021) and/or damage to the myodural bridges (Klinge et al., 2021; Labuda et al., 2022b; McElroy et al., 2019); also, it could result in compression of C2–C4 nerve roots which form a pain pathway serving the occipital dura (i.e., outer covering) of the brain (Allen et al., 2024; M. Garcia et al., 2022; Noseda et al., 2019). Interestingly, the chronic migraine-like headaches observed in approximately 60–80% of adult CMI patients occur in the occipital area (Fischbein et al., 2015). However, CMI patients initially show evidence of acute pressure, or Val Salva, headaches due to “water hammer” effects of the systolic heart cycle (i.e., a pressure wave on each cardiac cycle is blocked by cervico-medullary compression (Doberstein et al., 2017) but do not develop chronic migraine headaches until later in the progression of this syndrome. Approximately half of CMI patients undergo a surgical procedure known as cranial fossa decompression neurosurgery to alleviate cerebrospinal fluid (CSF) pressure in the cervico-medullary area. After recovery, CMI patients frequently show considerable improvement in Val Salva headaches but not in chronic occipital migraine headaches (Labuda et al., 2022a; Pepper et al., 2021). This evidence suggests that chronic headaches in CMI might potentially result from central sensitization of pain, whereas the Val Salva headaches in CMI are the result of nociceptive pain (Tokar et al., 2023).

## 9. Individual Differences in Personality Can Modulate Episodic Memory

It is noteworthy that the findings for pain in CM1 reviewed here suggest that emotional characteristics or affective modulation might influence the development of episodic memory in chronic pain syndromes. Negative affect can potentially mitigate the successful development of episodic representations—thereby ‘coloring’ or otherwise distracting from the allocation of executive resources to memory encoding. Accordingly, it is of considerable interest here to consider the interface between personality variables as emotional filters and/or modulators and the impact these traits might have on memory. What follows is a limited review of the evidence toward a more integrated perspective of pain, affect, and memory processing.

Research regarding personality characteristics offers unique insights in the cognitive-affective dimension of memory processing. Indeed, individual differences in personality traits, often measured using the NEO Personality Inventory (NEO-PI) (McCrae et al., 2005), include constructs with affect-related indicators such as neuroticism, extraversion, openness (to experience), conscientiousness, and agreeableness factors (i.e., the Big five personality factors). Not surprisingly, individual levels of these personality factors have been related to episodic memory performance. Using extreme-score analyses, high levels of conscientiousness and low levels of neuroticism, conscientiousness, extraversion, and openness are typically associated with better immediate and delayed recall. The apparent inverse association between neuroticism levels and episodic memory performance is particularly noteworthy (Allen et al., 2011; Chapman et al., 2017; Klaming et al., 2017; Luchetti et al., 2016). Clinically high levels of neuroticism have been associated with severe episodic memory decrements and also with higher morbidity and mortality rates (Huang et al., 2025; Kempen et al., 1997; Wilson et al., 2005). Somewhat complicating this relationship between neuroticism and memory are previous findings suggesting elevated normal levels of neuroticism are associated with enhanced episodic memory performance (Allen et al., 2005). Regardless of relational direction, it is reasonable to expect variations in affective tone (at the trait level) to influence episodic memory thus supporting the modulatory role of affect in cognitive information processing outcomes. One (Kempen et al., 1997) way to further explore the strength and direction of this modulation is to draw on established mechanisms and frameworks offered through the neuroscience perspective.

## 10. Affective Pain, Coping Mechanisms, and Acceptance and Commitment Therapy (ACT)

Chronic pain is especially disruptive to the encoding and retrieval of episodic memory (Allen et al., 2018; Eccleston & Crombez, 1999), highlighting the need to identify strategies that attenuate its interfering effects on episodic memory. One of the most widely used interventions for chronic pain is Acceptance and Commitment Therapy (ACT) (Trindade et al., 2021; Veehof et al., 2016; Wicksell et al., 2013). ACT is a contextual cognitive behavioral therapy that emphasizes both acceptance and mindfulness in addition to values and behavior change processes that promote well-being in a wide range of problem areas, including chronic pain in pain conditions like CMI (Hayes et al., 2011).

Garcia et al. conducted a CMI study with eight weeks of ACT therapy (*n* = 52) and found that both psychological flexibility and acceptance of chronic pain improved more rapidly in the ACT group than the wait-listed control group (M. A. Garcia et al., 2023). Rabinowitz and colleagues conducted a randomized clinical trial (*n* = 112) using eight sessions of online ACT in a population of CMI patients (Rabinowitz et al., 2024). This study included ACT + coaching, ACT only, and wait-listed controls. The ACT + coaching and ACT only groups showed significant improvement relative to wait-listed controls in chronic pain acceptance and anxiety reduction. While these three studies did not directly assess coping with chronic pain and episodic memory performance (although see (Allen et al., 2018)), the key ACT findings for CMI patients were better coping strategies with chronic pain.

## 11. Chronic Pain Inhibition, Episodic Memory and Chiari Malformation Type I

Given the potential impact of cognitive strategies on pain processing, it is instructive at this point to examine how accepting or inhibiting centrally sensitized pain in CMI may modulate episodic memory performance commonly observed in this condition (Allen et al., 2014; M. Garcia et al., 2018a, 2024; J. R. Houston et al., 2019; M. L. Houston et al., 2021; Rogers et al., 2018; Seaman et al., 2021). In a study examining a relatively large number of CMI patients (347 surgically decompressed individuals and 297 non-decompressed individuals, Allen and colleagues (Allen et al., 2018) tested participants on the Rumination and Reflection Questionnaire (RRQ (Trapnell & Campbell, 1999)) and the Rey Auditory Verbal Learning Task (RAVLT (Schmidt, 1996)). These investigators observed an association characterized by lower recall performance, both immediate and delayed, being associated with higher pain levels. RRQ reflection was associated with better recall on the RAVLT, whereas rumination was not related to recall or pain levels. It is important to note that RRQ reflection is an index of self-focused attention (Trapnell & Campbell, 1999). This study also showed that non-decompressed CMI patients with higher reflection scores and lower pain levels had higher delayed recall levels than decompressed CMI patients. At least two issues are noteworthy in these results. First, up to a point, some CMI patients (with high levels of self-focused attention) may have been able to inhibit pain so that they could encode and retrieve the RAVLT words more efficiently than individuals at higher levels of pain and lower levels of self-reflection. Second, this interaction between reflection level and pain for episodic recall occurred only in patients who had not undergone decompression surgery. It was hypothesized that this occurred because patients who had undergone neurosurgery experienced a placebo effect that acted as a buffer against affective pain. In other words, feeling that the surgery “fixed” them, they did not require the same attentional block of pain strategies that non-decompressed patients apparently utilized. Consequently, the core finding from this study was that as attentional inhibition of pain increased (as measured by individual differences in self-focused attention), episodic memory performance (as measured by RAVLT delayed recall) increased (Allen et al., 2018). This suggests that chronic pain interferes with attention resulting in distraction from episodic encoding or retrieval (Eccleston & Crombez, 1999), but that attentional suppression of pain can alleviate this effect. Incidentally, the presumed mechanisms underlying this buffering effect are similar to those targeted by ACT approaches as described previously (except in ACT theory the assumption is that individuals “accept” pain and in attentional inhibition that individuals “inhibit” pain). Perhaps these two mechanisms may be similar.

In addition to this study, there is other evidence that CMI involves episodic memory dysfunction. Indeed, at least five other studies on CMI and cognition have reported episodic memory deficits in this group (Allen et al., 2014; M. Garcia et al., 2018a, 2018b; J. R. Houston et al., 2019; Seaman et al., 2021). Consequently, there is considerable evidence that CMI involves episodic memory dysfunction, and that this dysfunction is associated with chronic pain. Also, there is evidence that ACT can help CMI patients better cope with pain (M. A. Garcia et al., 2023; Rabinowitz et al., 2024) but chronic pain requires constant selective attention filtering that is sensitive to coping resources (Al Samman et al., 2024; M. A. Garcia et al., 2021). This requires individuals with CMI to have enough psychological and biological coping mechanisms to deal with chronic pain due to external (e.g., loneliness) and internal (e.g., increased cortisol and anxiety) stressors. This issue is discussed in more detail in the next section.

## 12. The Role of Allostatic Load and Biological Resilience

Allostatic load reflects the biological (with an emphasis on hormonal) and psychological ‘wear and tear’ on one’s body (McEwen & Seeman, 1999). Chronic exposure to psychosocial and/or physical stressors (e.g., pain) can overwhelm one’s coping resources and result in allostatic overload (Juster et al., 2010; McEwen & Alves, 1999; McEwen & Stellar, 1993). In turn, allostatic overload is associated with pro-inflammatory results that can have adverse health (e.g., an increased risk of cardiovascular disease, cancer, dementia and Type II diabetes) and cognitive outcomes (Juster et al., 2010). With regard to CMI, there is evidence that higher levels of loneliness and functional disability result in a hypothalamic, pituitary, adrenal (HPA) axis response associated with interleukin-6 (IL-6) and cortisol dysregulation (M. A. Garcia et al., 2021). Also, the combined effects of psychological stressors (e.g., functional disability or loneliness) and biological stressors (e.g., anterior CSF space restriction) were shown to increase the levels of HPA axis response (e.g., IL-6) as well as the neuroprotective hormone estrogen (Al Samman et al., 2024). These results suggest that chronic pain in CMI can (and does) result in allostatic overload and can be reasonably implicated in CMI-related episodic memory dysfunction as measured by immediate and delayed recall (Allen et al., 2018) using a modified Rey Auditory Verbal Learning Task methodology (Schmidt, 1996). Consequently, these results also emphasize the potential value of chronic pain interventions that help build coping resources (e.g., higher levels of self-focused attention or lower levels of loneliness) to resist allostatic overload in addition to the type of inhibitory control buffering effect of therapy approaches such as ACT (Al Samman et al., 2024; Allen et al., 2018). Although pharmacological interventions can be problematic (see below), interventions that help build coping resources to resist allostatic overload have shown promise.

## 13. Pharmacological Treatments for Pain and the Effect on Memory and Cognition

Opioids (morphine, oxycodone, fentanyl) remain a cornerstone of chronic pain management, though long-term use has been associated with cognitive effects, including impairments in memory and attention, especially with increasing adult age (Warner et al., 2022; Yang et al., 2020). Opioid use for non-cancer pain treatment has been associated with the increase the risk of dementia and related brain-imaging abnormalities in large-scale studies (Lin et al., 2025). The paradigm of optimizing chronic pain management has mainly focused on reducing the addictive capacity of the drugs and less so to address cognitive sequalae and in this endeavor, most pain specialists now implement buprenorphine or naltrexone. Buprenorphine offers effective analgesia and lowers misuse risk—making it an excellent option for chronic pain management, especially in patients with complex comorbidities or prior opioid exposure. Because of its high receptor affinity and slow dissociation, buprenorphine can stabilize patients with opioid tolerance or dependence, offering analgesia without reinforcing addiction pathways (Spinella & McCarthy, 2024). Naltrexone is increasingly being explored and still used off-label as an adjunct in chronic pain management, but only in very low doses (so-called low-dose naltrexone, LDN). As a selective μ-opioid receptor blocker and microglial antagonist, it has growing use specifically in central sensitization syndromes (as discussed above) where inflammation and microglial activation play a key role (Dowell et al., 2022). It has been shown that both buprenorphine and naltrexone have fewer negative effects on cognition, besides other speculated context, mainly believed to act through lesser effects on sedation and respiratory depression and the subsequent cerebral hypoxia (Singh et al., 2021). When searching for cognitive associations, it has to be stressed that LDN has not been associated with any measurable cognitive or memory decline. In some reports, patients describe improved mental clarity and alertness, which researchers propose are secondary to: reduced neuroinflammation (microglial downregulation), improved sleep and reduced pain and fatigue (Bruun et al., 2024). For LDN, one study has shown no statistically significant superiority of LDN over placebo for pain; however, a significant improvement in memory problems was reported in the LDN group vs. placebo (Bruun et al., 2024). Experimental and clinical studies from two decades ago have indicated that memory recall or retrieval is modulated in the CA3 hippocampal mossy fiber layer (Meilandt et al., 2004). This layer carries many opioid receptors and, therefore, it is deemed to be the area of interest where memories associated with chronic pain and pharmacological pain treatment for pain intersect—proposed as such to be the basic mechanism for opioid antagonists and memory modulation. In support of this, a clinical trial has shown a significant effect of buprenorphine- and naloxone-related impairment in episodic/long-term memory (recognition memory) performance found only at the highest doses relative to the two lower doses (Mintzer et al., 2004). Also, as summarized in most resent overview evidence indicate that opioids may specifically impair episodic memory—especially in long-term or high-dose use (Bell et al., 2025).

Instead, NSAIDS have shown to modestly support cognitive function by lowering inflammatory mediators (IL-1β, TNF-α, PGE2), and unlike opioids, they do not cause central nervous system depression; thus, alertness, attention, and executive function were reported as generally preserved (W. J. Wang et al., 2016). Due to the lack of addiction, they are indeed recommended as first-line treatment for chronic non-cancer pain in WHO-adapted guidelines; however, long-term NSAID use is limited by gastrointestinal toxicity and consequent metabolic complications with dosing constraints in both pediatric and adult patients and, more importantly here, NSAIDs offer limited benefit for the predominantly central sensitization–driven pain (Moore et al., 2025), which leaves this option of limiting benefit for CM1 given the above proposed and outlined context of CM1 neuropathic and nociplastic pain and its effect on and association with episodic memory.

One can argue that much of the literature linking pain pharmacology to cognitive dysfunction stems from studies of opioids and their derivatives, suggesting that the negative cognitive effects may have been overstated. Modern approaches to chronic pain management as outlined above have evolved to mitigate these concerns and may be less detrimental to cognition than previously thought. Besides this, it has also been recognized that psychologically reducing the subjective experience of pain can alleviate cognitive sequelae, likely by lessening cognitive load and limbic hyperactivation associated with chronic pain (Cunningham et al., 2019). Together, these findings indicate that effective, proactive pain management may not only improve quality of life but also help preserve cognitive function and memory in patients with chronic pain. However, the pain literature in CM1 also indicates that improved pain control includes psychological interventions needing emotional and behavioral control which circles back to the factoring in of personal traits and coping mechanisms in the modulation of episodic memory and cognition in chronic pain.as discussed in our review (Cunningham et al., 2019). The benefit of coping strategies in modifying pharmacological treatment in relation to cognition is an area of interest.

The optimization of pharmacological management of pain and outcome in Chiari malformation is evolving, with a growing emphasis on non-opioid and adjunctive therapies using neuromodulation medication, such as gabapentin and pregabalin (Barpujari et al., 2023). Gabapentin is particularly useful for centralized pain conditions, such as nociplastic pain in fibromyalgia, with lower addiction potential compared to opioids and fewer gastrointestinal complications than NSAIDS, making it an attractive option from CM1. Gabepentin and derivatives also have an anxiolytic effect, however, by decreasing excitatory neurotransmitter release and broadly dampening neuronal signaling, including hippocampal pathways—they can impair cognition (Behroozi et al., 2023): they have shown to affect working memory rather than episodic memory, as observed with opioids. Gabapentin has further negatively impacted mood and motivation and contributed to apathy and depression; depression is truly relevant to CM1-related cognitive issues, as extensively discussed above, and therefore may exacerbate the problem rather than mitigating it. These effects are dose-dependent and represent meaningful cognitive and motivational side effects in CM1 patients (Behroozi et al., 2023). Another concern recently linked to Gabapentin and derivates is the unclear modulation of neuroprotective microglia impairing potentially beneficial microglial functions (Cavalcanti et al., 2025).

In summary, a tailored, multimodal approach is crucial to effectively address the complex pain syndromes associated with CM1. Studies addressing pharmacological management in CM1 in both adult and pediatric patients address pain treatment and its benefits mainly in conjunction with surgical decompression (Lu et al., 2021); however, the dual modality has shown to improve functional outcomes mainly in pediatric CM1. To date, research linking pharmacological interventions to cognitive performance in CM1 is limited and more research is encouraged.

## 14. Conclusions

Episodic memory is contextual memory linking temporal, spatial, emotional, and self-referential contexts. In this review, we placed particular emphasis on the emotional context. These contextual markers are useful for memory retrieval. We discussed a somatic marker model of emotional contexts and reviewed the literature on how affect-associated individual differences in personality variables (e.g., conscientiousness, openness to experience, extraversion, and neuroticism) could be used to bolster episodic memory. However, the world we live in is replete with stressors that require selective attention for focusing on pertinent targets and filtering (inhibiting) non-pertinent targets. As such, we reviewed how chronic pain was an especially challenging prepotent distractor and used a chronic pain syndrome, Chiari malformation Type I (CMI), as an example of how pain could affect episodic memory performance. Lastly, we discussed the importance of managing allostatic load to avoid the negative impact of overload on well-being and episodic memory function, as well as the risks for memory impairment and dementia from using long-term opiate treatments for chronic pain.

In terms of future directions, because of the prevalence of chronic pain, we need more research on how chronic pain affects episodic memory performance. One potential method of doing this is to collect EEG data when a recognition memory task (old/new) is being presented after a delay (retention interval) before and after posterior cranial fossa decompression surgery in Chiari patients and across the same time period in age- and education-matched controls. This approach has been used to study recognition memory in individuals diagnosed with mild cognitive impairment (Y. Y. Wang et al., 2023). Also, brain stimulation (e.g., entrainment) and inhibition (e.g., transcranial stimulation, or TMS treatment) may provide future avenues for improving episodic memory performance. Some evidence does suggest that repetitive TMS treatment does attenuate chronic pain (Hamid et al., 2019).

## Data Availability

No new data were created or analyzed in this study. Data sharing is not applicable to this article.

## References

[B1-behavsci-15-01678] Alexandra Kredlow M., Fenster R. J., Laurent E. S., Ressler K. J., Phelps E. A. (2022). Prefrontal cortex, amygdala, and threat processing: Implications for PTSD. Neuropsychopharmacology.

[B2-behavsci-15-01678] Aliaga L., Hekman K. E., Yassari R., Straus D., Luther G., Chen J., Sampat A., Frim D. (2012). A novel scoring system for assessing Chiari malformation type I treatment outcomes. Neurosurgery.

[B3-behavsci-15-01678] Allen P. A., Delahanty D., Kaut K. P., Li X., Garcia M., Houston J. R., Tokar D. M., Loth F., Maleki J., Vorster S., Luciano M. G. (2018). Chiari 1000 registry project: Assessment of surgical outcome on self-focused attention, pain, and delayed recall. Psychological Medicine.

[B4-behavsci-15-01678] Allen P. A., Houston J. R., Pollock J. W., Buzzelli C., Li X., Harrington A. K., Martin B. A., Loth F., Lien M. C., Maleki J., Luciano M. G. (2014). Task-specific and general cognitive effects in Chiari malformation type I. PLoS ONE.

[B5-behavsci-15-01678] Allen P. A., Hughes M. L., Houston J. R., Jardin E., Mallik P., McLennan C., Delahanty D. L. (2019). Are there age differences in consolidated episodic memory?. Experimental Aging Research.

[B6-behavsci-15-01678] Allen P. A., Kaut K., Baena E., Lien M. C., Ruthruff E. (2011). Individual differences in positive affect moderate age-related declines in episodic long-term memory. Journal of Cognitive Psychology.

[B7-behavsci-15-01678] Allen P. A., Kaut K. P., Lord R. G., Hall R. J., Grabbe J. W., Bowie T. (2005). An emotional mediation theory of differential age effects in episodic and semantic memories. Experimental Aging Research.

[B8-behavsci-15-01678] Allen P. A., Kaut K. P., Lord R. R. (2008). Emotion and episodic memory. Handbook of episodic memory.

[B9-behavsci-15-01678] Allen P. A., Loth F., Loth D., Samman M. M. A., Labuda R., Herrera C., Bapuraj J. R., Klinge P. M. (2024). Correlation of anterior CSF space in the cervical spine with Chicago Chiari Outcome Scale score in adult females. Journal of Neurosurgery-Spine.

[B10-behavsci-15-01678] Al Samman M. M., Garcia M. A., Garcia M., Houston J. R., Loth D., Labuda R., Vorster S., Klinge P. M., Loth F., Delahanty D. L., Allen P. A. (2024). Relationship of morphometrics and symptom severity in female type I Chiari malformation patients with biological resilience. The Cerebellum.

[B11-behavsci-15-01678] Aly M., Turk-Browne N. B. (2016). Attention promotes episodic encoding by stabilizing hippocampal representations. Proceedings of the National Academy of Sciences of the United States of America.

[B12-behavsci-15-01678] Barpujari A., Kiley A., Ross J. A., Veznedaroglu E. (2023). A systematic review of non-opioid pain management in Chiari malformation (type 1) patients: Current evidence and novel therapeutic opportunities. Journal of Clinical Medicine.

[B13-behavsci-15-01678] Bechara A., Damasio H., Tranel D., Damasio A. R. (2005). The Iowa gambling task and the somatic marker hypothesis: Some questions and answers. Trends in Cognitive Sciences.

[B14-behavsci-15-01678] Behroozi Z., Jafarpour M., Razmgir M., Saffarpour S., Azizi H., Kheirandish A., Kosari-rad T., Ramezni F., Janzadeh A. (2023). The effect of gabapentin and pregabalin administration on memory in clinical and preclinical studies: A meta-analysis and systematic review. BMC Psychiatry.

[B15-behavsci-15-01678] Bell T. R., Elman J. A., Gustavson D. E., Lyons M. J., Fennema-Notestine C., Williams M. E., Panizzon M. S., Pearce R. C., Reynolds C. A., Sanderson-Cimino M., Toomey R., Jak A., Franz C. E., Kremen W. S. (2025). History of chronic pain and opioid use is associated with cognitive decline and mild cognitive impairment. Journal of the International Neuropsychological Society.

[B16-behavsci-15-01678] Bertossi E., Aleo F., Braghittoni D., Ciaramelli E. (2016). Stuck in the here and now: Construction of fictitious and future experiences following ventromedial prefrontal damage. Neuropsychologia.

[B17-behavsci-15-01678] Bower G. H. (1981). Mood and memory. American Psychologist.

[B18-behavsci-15-01678] Brown R., Kulik J. (1977). Flashbulb memories. Cognition.

[B19-behavsci-15-01678] Bruun K. D., Christensen R., Amris K., Vaegter H. B., Blichfeldt-Eckhardt M. R., Bye-Moller L., Holsgaard-Larsen A., Toft P. (2024). Naltrexone 6 mg once daily versus placebo in women with fibromyalgia: A randomised, double-blind, placebo-controlled trial. Lancet Rheumatology.

[B20-behavsci-15-01678] Cabeza R. (2001). Cognitive neuroscience of aging: Contributions of functional neuroimaging. Scandinavian Journal of Psychology.

[B21-behavsci-15-01678] Cabeza R. (2002). Hemispheric asymmetry reduction in older adults: The HAROLD model. Psychology and Aging.

[B22-behavsci-15-01678] Calabrese P., Markowitsch H. J., Durwen H. F., Widlitzek H., Haupts M., Holinka B., Gehlen W. (1996). Right temporofrontal cortex as critical locus for the ecphory of old episodic memories. Journal of Neurology Neurosurgery and Psychiatry.

[B23-behavsci-15-01678] Cavalcanti R. R., Almeida F. M., Martinez A. M. B., Freria C. M. (2025). Neuroinflammation: Targeting microglia for neuroprotection and repair after spinal cord injury. Frontiers in Immunology.

[B24-behavsci-15-01678] Chapman B. P., Benedict R. H., Lin F., Roy S., Federoff H. J., Mapstone M. (2017). Personality and performance in specific neurocognitive domains among older persons. American Journal of Geriatric Psychiatry.

[B25-behavsci-15-01678] Clarke E. C., Fletcher D. F., Stoodley M. A., Bilston L. E. (2013a). Computational fluid dynamics modelling of cerebrospinal fluid pressure in Chiari malformation and syringomyelia. Journal of Biomechanics.

[B26-behavsci-15-01678] Clarke E. C., Stoodley M. A., Bilston L. E. (2013b). Changes in temporal flow characteristics of CSF in Chiari malformation type I with and without syringomyelia: Implications for theory of syrinx development Clinical article. Journal of Neurosurgery.

[B27-behavsci-15-01678] Corkin S. (1984). Lasting consequences of bilateral medial temporal lobectomy: Clinical course and experimental findings in H.M. Seminars in Neurology.

[B28-behavsci-15-01678] Cunningham N. R., Kashikar-Zuck S., Coghill R. C. (2019). Brain mechanisms impacted by psychological therapies for pain: Identifying targets for optimization of treatment effects. Pain Reports.

[B29-behavsci-15-01678] Damasio A. R. (1994). Descartes’ error: Emotion, reason, and the human brain.

[B30-behavsci-15-01678] Davies R. R., Graham K. S., Xuereb J. H., Williams G. B., Hodges J. R. (2004). The human perirhinal cortex and semantic memory. European Journal of Neuroscience.

[B31-behavsci-15-01678] Demonet J. F., Celsis P., Nespoulous J. L., Viallard G., Marcvergnes J. P., Rascol A. (1992). Cerebral blood-flow correlates of word monitoring in sentences—Influence of semantic incoherence—A spect study in normals. Neuropsychologia.

[B32-behavsci-15-01678] D’Esposito M. D., Postle B. R., Stuss D. T., Knight R. T. (2002). The organization of working memory function in lateral prefrontal cortex: Evidence from event-related functional MRI. Principles of frontal lobe function.

[B33-behavsci-15-01678] Dew I. T. Z., Cabeza R. (2011). The porous boundaries between explicit and implicit memory: Behavioral and neural evidence. Annals of the New York Academy of Sciences.

[B34-behavsci-15-01678] Doberstein C. A., Torabi R., Klinge P. M. (2017). Current concepts in the pathogenesis, diagnosis, and management of type I Chiari malformations. Rhode Island Medical Journal (2013).

[B35-behavsci-15-01678] Dowell D., Ragan K. R., Jones C. M., Baldwin G. T., Chou R. (2022). Prescribing opioids for pain—The new CDC clinical practice guideline. New England Journal of Medicine.

[B36-behavsci-15-01678] Dworkin R. H., Turk D. C., Revicki D. A., Harding G., Coyne K. S., Peirce-Sandner S., Bhagwat D., Everton D., Burke L. B., Cowan P., Farrar J. T. (2009). Development and initial validation of an expanded and revised version of the Short-form McGill Pain Questionnaire (SF-MPQ-2). Pain.

[B37-behavsci-15-01678] Eccleston C., Crombez G. (1999). Pain demands attention: A cognitive-affective model of the interruptive function of pain. Psychological Bulletin.

[B38-behavsci-15-01678] Eppelheimer M. S., Houston J. R., Bapuraj J. R., Labuda R., Loth D. M., Braun A. M., Allen N. J., Heidari Pahlavian S., Biswas D., Urbizu A., Martin B. A., Maher C. O., Allen P. A., Loth F. (2018). A retrospective 2D morphometric analysis of adult female Chiari type I patients with commonly reported and related conditions. Frontiers in Neuroanatomy.

[B39-behavsci-15-01678] Fabri T. L., Datta R., O’Mahony J., Barlow-Krelina E., De Somma E., Longoni G., Gur R. E., Gur R. C., Bacchus M., Yeh E. A., Banwell B. L., Till C. (2021). Memory, processing of emotional stimuli, and volume of limbic structures in pediatric-onset multiple sclerosis. Neuroimage-Clinical.

[B40-behavsci-15-01678] Fischbein R., Saling J. R., Marty P., Kropp D., Meeker J., Amerine J., Chyatte M. R. (2015). Patient-reported Chiari malformation type I symptoms and diagnostic experiences: A report from the national Conquer Chiari Patient Registry database. Neurological Sciences.

[B41-behavsci-15-01678] Friedlander R. M. (2024). Congenital and acquired Chiari syndrome. New England Journal of Medicine.

[B42-behavsci-15-01678] Garcia M., Amayra I., Lazaro E., Lopez-Paz J. F., Martinez O., Perez M., Berrocoso S., Al-Rashaida M. (2018a). Comparison between decompressed and non-decompressed Chiari malformation type I patients: A neuropsychological study. Neuropsychologia.

[B43-behavsci-15-01678] Garcia M., Amayra I., Pérez M., Salgueiro M., Martínez O., López-Paz J. F., Allen P. A. (2024). Cognition in Chiari malformation type I: An update of a systematic review. Neuropsychology Review.

[B44-behavsci-15-01678] Garcia M., Eppelheimer M. S., Houston J. R., Houston M. L., Nwotchouang B. S. T., Kaut K. P., Labuda R., Bapuraj J. R., Maleki J., Klinge P. M., Vorster S., Luciano M. G., Loth F., Allen P. A. (2022). Adult age differences in self-reported pain and anterior CSF space in Chiari malformation. Cerebellum.

[B45-behavsci-15-01678] Garcia M., Lazaro E., Lopez-Paz J. F., Martinez O., Perez M., Berrocoso S., Al-Rashaida M., Amayra I. (2018b). Cognitive functioning in Chiari malformation type I without posterior fossa surgery. Cerebellum.

[B46-behavsci-15-01678] Garcia M. A., Allen P. A., Li X., Houston J. R., Loth F., Labuda R., Delahanty D. L. (2019). An examination of pain, disability, and the psychological correlates of Chiari malformation pre- and post-surgical correction. Disability and Health Journal.

[B47-behavsci-15-01678] Garcia M. A., Li X., Allen P. A., Delahanty D. L., Eppelheimer M. S., Houston J. R., Johnson D. M., Loth F., Maleki J., Vorster S., Luciano M. G. (2021). Impact of surgical status, loneliness, and disability on interleukin 6, C-reactive protein, cortisol, and estrogen in females with symptomatic type I Chiari malformation. The Cerebellum.

[B48-behavsci-15-01678] Garcia M. A., Rabinowitz E. P., Levin M. E., Shasteen H., Allen P. A., Delahanty D. L. (2023). Online acceptance and commitment therapy for chronic pain in a sample of people with Chiari malformation: A pilot study. Journal of Behavioral and Cognitive Therapy.

[B49-behavsci-15-01678] Gholampour S., Gholampour H. (2020). Correlation of a new hydrodynamic index with other effective indexes in Chiari I malformation patients with different associations. Scientific Reports.

[B50-behavsci-15-01678] Gholampour S., Taher M. (2018). Relationship of morphologic changes in the brain and spinal cord and disease symptoms with cerebrospinal fluid hydrodynamic changes in patients with Chiari malformation type I. World Neurosurgery.

[B51-behavsci-15-01678] Glanzer M., Bower G. H., Spence J. T. (1972). Storage mechanisms in recall. The psychology of learning and motivation.

[B52-behavsci-15-01678] Goel A. (2015). Is atlantoaxial instability the cause of Chiari malformation? Outcome analysis of 65 patients treated by atlantoaxial fixation. Journal of Neurosurgery: Spine.

[B53-behavsci-15-01678] Goel A., Jadhav D., Shah A., Rai S., Dandpat S., Vutha R., Dhar A., Prasad A. (2020). Chiari 1 formation redefined-clinical and radiographic observations in 388 surgically treated patients. World Neurosurgery.

[B54-behavsci-15-01678] Hamid P., Malik B. H., Hussain M. L. (2019). Noninvasive transcranial magnetic stimulation (TMS) in chronic refractory pain: A systematic review. Cureus.

[B55-behavsci-15-01678] Hayes S. C., Villatte M., Levin M., Hildebrandt M. (2011). Open, aware, and active: Contextual approaches as an emerging trend in the behavioral and cognitive therapies. Annual Review of Clinical Psychology.

[B56-behavsci-15-01678] Heiss J. D. (2023). Cerebrospinal fluid hydrodynamics in Chiari I malformation and syringomyelia: Modeling pathophysiology. Neurosurgery Clinics of North America.

[B57-behavsci-15-01678] Hodges J. R., Patterson K. (1997). Semantic memory disorders. Trends in Cognitive Sciences.

[B58-behavsci-15-01678] Houston J. R., Allen P. A., Rogers J. M., Lien M. C., Allen N. J., Hughes M. L., Bapuraj J. R., Eppelheimer M. S., Loth F., Stoodley M. A., Vorster S. J., Luciano M. G. (2019). Type I Chiari malformation, RBANS performance, and brain morphology: Connecting the dots on cognition and macrolevel brain structure. Neuropsychology.

[B59-behavsci-15-01678] Houston J. R., Eppelheimer M. S., Pahlavian S. H., Biswas D., Urbizu A., Martin B. A., Bapuraj J. R., Luciano M., Allen P. A., Loth F. (2018). A morphometric assessment of type I Chiari malformation above the McRae line: A retrospective case-control study in 302 adult female subjects. Journal of Neuroradiology.

[B60-behavsci-15-01678] Houston J. R., Hughes M. L., Bennett I. J., Allen P. A., Rogers J. M., Lien M. C., Stoltz H., Sakaie K., Loth F., Maleki J., Vorster S. J., Luciano M. G. (2020). Evidence of neural microstructure abnormalities in type I Chiari malformation: Associations among fiber tract integrity, pain, and cognitive dysfunction. Pain Medicine.

[B61-behavsci-15-01678] Houston J. R., Maleki J., Loth F., Klinge P. M., Allen P. A. (2022). Influence of pain on cognitive dysfunction and emotion dysregulation in Chiari malformation type I.

[B62-behavsci-15-01678] Houston M. L., Houston J. R., Sakaie K., Klinge P. M., Vorster S., Luciano M., Loth F., Allen P. A. (2021). Functional connectivity abnormalities in type I Chiari: Associations with cognition and pain. Brain Communications.

[B63-behavsci-15-01678] Huang S., Zhang Y., Ma L., Wu B., Feng J., Cheng W., Yu J. (2025). Neuroticism is associated with future disease and mortality risks. Chinese Medical Journal.

[B64-behavsci-15-01678] Ibrahimy A., Wu T. X., Mack J., Scott G. C., Cortes M. X., Cantor F. K., Loth F., Heiss J. D. (2023). Prospective, longitudinal study of clinical outcome and morphometric posterior fossa changes after craniocervical decompression for symptomatic Chiari I malformation. American Journal of Neuroradiology.

[B65-behavsci-15-01678] Ikeda M., Mori E., Hirono N., Imamura T., Shimomura T., Ikejiri Y., Yamashita H. (1998). Amnestic people with Alzheimer’s disease who remembered the Kobe earthquake. British Journal of Psychiatry.

[B66-behavsci-15-01678] Juster R. P., McEwen B. S., Lupien S. J. (2010). Allostatic load biomarkers of chronic stress and impact on health and cognition. Neuroscience & Biobehavioral Reviews.

[B67-behavsci-15-01678] Kamali A., Karbasian N., Rabiei P., Cano A., Riascos R. F., Tandon N., Arevalo O., Ocasio L., Younes K., Khayat-khoei M., Mirbagheri S., Hasan K. M. (2018). Revealing the cerebello-ponto-hypothalamic pathway in the human brain. Neuroscience Letters.

[B68-behavsci-15-01678] Kamali A., Milosavljevic S., Gandhi A., Lano K. R., Shobeiri P., Sherbaf F. G., Sair H. I., Riascos R. F., Hasan K. M. (2023). The Cortico-Limbo-Thalamo-Cortical circuits: An update to the original papez circuit of the human limbic system. Brain Topography.

[B69-behavsci-15-01678] Kapur N., Ellison D., Parkin A. J., Hunkin N. M., Burrows E., Sampson S. A., Morrison E. A. (1994). Bilateral temporal-lobe pathology with sparing of medial temporal-lobe structures—Lesion profile and pattern of memory disorder. Neuropsychologia.

[B70-behavsci-15-01678] Kartsounis L. D., Rudge P., Stevens J. M. (1995). Bilateral lesions of Ca1 and Ca2 fields of the hippocampus are sufficient to cause a severe amnesic syndrome in humans. Journal of Neurology Neurosurgery and Psychiatry.

[B71-behavsci-15-01678] Kempen G. I., Jelicic M., Ormel J. (1997). Personality, chronic medical morbidity, and health-related quality of life among older persons. Health Psychology.

[B72-behavsci-15-01678] Kensinger E. A. (2009). Emotional memory across the adult lifespan.

[B73-behavsci-15-01678] Kilpatrick L., Cahill L. (2003). Amygdala modulation of parahippocampal and frontal regions during emotionally influenced memory storage. Neuroimage.

[B74-behavsci-15-01678] Klaming R., Veltman D. J., Comijs H. C. (2017). The impact of personality on memory function in older adults-results from the longitudinal aging study Amsterdam. International Journal of Geriatric Psychiatry.

[B75-behavsci-15-01678] Klinge P. M., McElroy A., Donahue J. E., Brinker T., Gokaslan Z. L., Beland T. B. (2021). Abnormal spinal cord motion at the craviocervical junction in hypermobile Ehlers-Danlos patients. Journal of Neurosurgery-Spine.

[B76-behavsci-15-01678] LaBar K. S., Cabeza R. (2006). Cognitive neuroscience of emotional memory. Nature Reviews Neuroscience.

[B77-behavsci-15-01678] LaBar K. S., Phelps E. A. (1998). Arousal-mediated memory consolidation: Role of the medial temporal lobe in humans. Psychological Science.

[B78-behavsci-15-01678] Labuda R., Loth D., Allen P. A., Loth F. (2022a). Factors associated with patient-reported postsurgical symptom improvement in adult females with Chiari malformation type I: A report from the Chiari1000 dataset. World Neurosurgery.

[B79-behavsci-15-01678] Labuda R., Nwotchouang B. S. T., Ibrahimy A., Allen P. A., Oshinski J. N., Klinge P., Loth F. (2022b). A new hypothesis for the pathophysiology of symptomatic adult Chiari malformation type I. Medical Hypotheses.

[B80-behavsci-15-01678] Langley L. K., Madden D. J. (2000). Functional neuroimaging of memory: Implications for cognitive aging. Microscopy Research and Technique.

[B81-behavsci-15-01678] Leigland L. A., Schulz L. E., Janowsky J. S. (2004). Age related changes in emotional memory. Neurobiology of Aging.

[B82-behavsci-15-01678] Leonard J. R., Limbrick D. D. (2015). Chiari I malformation: Adult and pediatric considerations. Neurosurgery Clinics of North America.

[B83-behavsci-15-01678] Leung V., Magnussen J. S., Stoodley M. A., Bilston L. E. (2016). Cerebellar and hindbrain motion in Chiari malformation with and without syringomyelia. Journal of Neurosurgery-Spine.

[B84-behavsci-15-01678] Levenson R. W., Carstensen L. L., Gottman J. M. (1994). The influence of age and gender on affect, physiology, and their interrelations—A study of long-term marriages. Journal of Personality and Social Psychology.

[B85-behavsci-15-01678] Levenson R. W., Friesen W. V., Ekman P., Carstensen L. L. (1991). Emotion, physiology, and expression in old-age. Psychology and Aging.

[B86-behavsci-15-01678] Lin T., Barash J. A., Wang S., Li F., Yang Z., Kofke W. A., Sha F., Tang J. (2025). Regular use of opioids and dementia, cognitive measures, and neuroimaging outcomes among UK Biobank participants with chronic non-cancer pain. Alzheimer’s & Dementia.

[B87-behavsci-15-01678] Lu V. M., Daniels D. J., Haile D. T., Ahn E. S. (2021). Effects of intraoperative liposomal bupivacaine on pain control and opioid use after pediatric Chiari I malformation surgery: An initial experience. Journal of Neurosurgery-Pediatrics.

[B88-behavsci-15-01678] Luchetti M., Terracciano A., Stephan Y., Sutin A. R. (2016). Personality and cognitive decline in older adults: Data from a longitudinal sample and meta-analysis. Journals of Gerontology Series B-Psychological Sciences and Social Sciences.

[B89-behavsci-15-01678] Marsh E. J., Roediger H. L., Healy A. F., Proctor R. W., Weiner I. B. (2013). Episodic and autobiographical memory. Handbook of psychology: Experimental psychology.

[B90-behavsci-15-01678] McCormick C., Ciaramelli E., De Luca F., Maguire E. A. (2018). Comparing and contrasting the cognitive effects of hippocampal and ventromedial prefrontal cortex damage: A review of human lesion studies. Neuroscience.

[B91-behavsci-15-01678] McCrae R. R., Costa P. T., Martin T. A. (2005). The NEO-PI-3: A more readable revised NEO Personality Inventory. Journal of Personality Assessment.

[B92-behavsci-15-01678] McElroy A., Rashmir A., Manfredi J., Sledge D., Carr E., Stopa E., Klinge P. (2019). Evaluation of the structure of myodural bridges in an equine model of ehlers-danlos syndromes. Scientific Reports.

[B93-behavsci-15-01678] McEwen B. S., Alves S. E. (1999). Estrogen actions in the central nervous system. Endocrine Reviews.

[B94-behavsci-15-01678] McEwen B. S., Seeman T. (1999). Protective and damaging effects of mediators of stress—Elaborating and testing the concepts of allostasis and allostatic load. Socioeconomic Status and Health in Industrial Nations.

[B95-behavsci-15-01678] McEwen B. S., Stellar E. (1993). Stress and the individual: Mechanisms leading to disease. Archives of Internal Medicine.

[B96-behavsci-15-01678] McGaugh J. L. (2000). Memory—A century of consolidation. Science.

[B97-behavsci-15-01678] Meilandt W. J., Barea-Rodriguez E., Harvey S. A. K., Martinez J. (2004). Role of hippocampal CA3 μ-opioid receptors in spatial learning and memory. Journal of Neuroscience.

[B98-behavsci-15-01678] Melo B., Winocur G., Moscovitch M. (1999). False recall and false recognition: An examination of the effects of selective and combined lesions to the medial temporal lobe diencephalon and frontal lobe structures. Cognitive Neuropsychology.

[B99-behavsci-15-01678] Melzack R., Wall P. D. (1965). Pain mechanisms: A new theory. Science.

[B100-behavsci-15-01678] Mendell L. M. (2014). Constructing and deconstructing the gate theory of pain. Pain.

[B101-behavsci-15-01678] Menon V. (2023). 20 years of the default mode network: A review and synthesis. Neuron.

[B102-behavsci-15-01678] Menon V., Boyett-Anderson J. M., Schatzberg A. F., Reiss A. L. (2002). Relating semantic and episodic memory systems. Cognitive Brain Research.

[B103-behavsci-15-01678] Milhorat T. H., Chou M. W., Trinidad E. M., Kula R. W., Mandell M., Wolpert C., Speer M. C. (1999). Chiari I malformation redefined: Clinical and radiographic findings for 364 symptomatic patients. Neurosurgery.

[B104-behavsci-15-01678] Mintzer M. Z., Correia C. J., Strain E. C. (2004). A dose-effect study of repeated administration of buprenorphine/naloxone on performance in opioid-dependent volunteers. Drug and Alcohol Dependence.

[B105-behavsci-15-01678] Moore A., Bidonde J., Fisher E., Häuser W., Bell R. F., Perrot S., Makri S., Straube S. (2025). Effectiveness of pharmacological therapies for fibromyalgia syndrome in adults: An overview of cochrane reviews. Rheumatology.

[B106-behavsci-15-01678] Nadel L., Moscovitch M. (1998). Hippocampal contributions to cortical plasticity. Neuropharmacology.

[B107-behavsci-15-01678] Noseda R., Melo-Carrillo A., Nir R.-R., Strassman A. M., Burstein R. (2019). Non-trigeminal nociceptive innervation of the posterior dura: Implications to occipital headache. The Journal of Neuroscience.

[B108-behavsci-15-01678] Pepper J., Elhabal A., Tsermoulas G., Flint G. (2021). Symptom outcome after craniovertebral decompression for Chiari type 1 malformation without syringomyelia. Acta Neurochirurgica.

[B109-behavsci-15-01678] Petersen S. E., Fox P. T., Posner M. I., Mintun M., Raichle M. E. (1988). Positron emission tomographic studies of the cortical anatomy of single-word processing. Nature.

[B110-behavsci-15-01678] Peterson L. R., Peterson M. J. (1959). Short-term retention of individual verbal items. Journal of Experimental Psychology.

[B111-behavsci-15-01678] Posner M. I., Petersen S. E., Fox P. T., Raichle M. E. (1988). Localization of cognitive operations in the human-brain. Science.

[B112-behavsci-15-01678] Rabinowitz E. P., Ripley G., Levin M. E., Allen P. A., Delahanty D. L. (2024). Limited effects of phone coaching in an RCT of online self-guided acceptance and commitment therapy for chronic pain. Journal of Contextual Behavioral Science.

[B113-behavsci-15-01678] Reed J. M., Squire L. R. (1997). Impaired recognition memory in patients with lesions limited to the hippocampal formation. Behavioral Neuroscience.

[B114-behavsci-15-01678] Reed J. M., Squire L. R. (1998). Retrograde amnesia for facts and events: Findings from four new cases. Journal of Neuroscience.

[B115-behavsci-15-01678] RempelClower N. L., Zola S. M., Squire L. R., Amaral D. G. (1996). Three cases of enduring memory impairment after bilateral damage limited to the hippocampal formation. Journal of Neuroscience.

[B116-behavsci-15-01678] Rogers J. M., Savage G., Stoodley M. A. (2018). A systematic review of cognition in Chiari I malformation. Neuropsychology Review.

[B117-behavsci-15-01678] Schmidt M. (1996). Rey auditory verbal learning test: RAVLT: A handbook.

[B118-behavsci-15-01678] Seaman S. C., Streese C. D., Manzel K., Kamm J., Menezes A. H., Tranel D., Dlouhy B. J. (2021). Cognitive and psychological functioning in Chiari malformation type I before and after surgical decompression—A prospective cohort study. Neurosurgery.

[B119-behavsci-15-01678] Shapira-Lichter I., Oren N., Jacob Y., Gruberger M., Hendler T. (2013). Portraying the unique contribution of the default mode network to internally driven mnemonic processes. Proceedings of the National Academy of Sciences of the United States of America.

[B120-behavsci-15-01678] Sharot T., Delgado M. R., Phelps E. A. (2004). How emotion enhances the feeling of remembering. Nature Neuroscience.

[B121-behavsci-15-01678] Singh A., Rao R., Chatterjee B., Mishra A. K., Kaloiya G., Ambekar A. (2021). Cognitive functioning in patients maintained on buprenorphine at peak and trough buprenorphine levels: An experimental study. Asian Journal of Psychiatry.

[B122-behavsci-15-01678] Spinella S., McCarthy R. (2024). Buprenorphine for pain: A narrative review and practical applications. The American Journal of Medicine.

[B123-behavsci-15-01678] Squire L. R. (2004). Memory systems of the brain: A brief history and current perspective. Neurobiology of Learning and Memory.

[B124-behavsci-15-01678] Squire L. R., Zolamorgan S. (1991). The medial temporal-lobe memory system. Science.

[B125-behavsci-15-01678] Teng E., Squire L. R. (1999). Memory for places learned long ago is intact after hippocampal damage. Nature.

[B126-behavsci-15-01678] Teyler T. J., Discenna P. (1986). The hippocampal memory indexing theory. Behavioral Neuroscience.

[B127-behavsci-15-01678] Tokar D. M., Kaut K. P., Allen P. A. (2023). Revisiting the factor structure of the Short-Form McGill Pain Questionnaire-2 (SF-MPQ-2): Evidence for a bifactor model in individuals with Chiari malformation. PLoS ONE.

[B128-behavsci-15-01678] Trapnell P. D., Campbell J. D. (1999). Private self-consciousness and the five-factor model of personality: Distinguishing rumination from reflection. Journal of Personality and Social Psychology.

[B129-behavsci-15-01678] Trindade I. A., Guiomar R., Carvalho S. A., Duarte J., Lapa T., Menezes P., Nogueira M. R., Patrao B., Pinto-Gouveia J., Castilho P. (2021). Efficacy of online-based acceptance and commitment therapy for chronic pain: A systematic review and meta-analysis. Journal of Pain.

[B130-behavsci-15-01678] Tulving E. (1983). Elements of episodic memory. Oxford psychology series no. 2.

[B131-behavsci-15-01678] Tulving E. (1985). Memory and consciousness. Canadian Psychology-Psychologie Canadienne.

[B132-behavsci-15-01678] VarghaKhadem F., Gadian D. G., Watkins K. E., Connelly A., VanPaesschen W., Mishkin M. (1997). Differential effects of early hippocampal pathology on episodic and semantic memory. Science.

[B133-behavsci-15-01678] Veehof M. M., Trompetter H. R., Bohlmeijer E. T., Schreurs K. M. G. (2016). Acceptance- and mindfulness-based interventions for the treatment of chronic pain: A meta-analytic review. Cognitive Behaviour Therapy.

[B134-behavsci-15-01678] Wang W. J., Sun Y. P., Zhang D. F. (2016). Association between non-steroidal anti-inflammatory drug use and cognitive decline: A systematic review and meta-analysis of prospective cohort studies. Drugs & Aging.

[B135-behavsci-15-01678] Wang Y. Y., Ye X., Song B., Yan Y. X., Ma W. Y., Shi J. P. (2023). Features of event-related potentials during retrieval of episodic memory in patients with mild cognitive impairment due to Alzheimer’s disease. Frontiers in Neuroscience.

[B136-behavsci-15-01678] Warner N. S., Hanson A. C., Schulte P. J., Habermann E. B., Warner D. O., Mielke M. M. (2022). Prescription opioids and longitudinal changes in cognitive function in older adults: A population-based observational study. Journal of the American Geriatrics Society.

[B137-behavsci-15-01678] Wicksell R. K., Kemani M., Jensen K., Kosek E., Kadetoff D., Sorjonen K., Ingvar M., Olsson G. L. (2013). Acceptance and commitment therapy for fibromyalgia: A randomized controlled trial. European Journal of Pain.

[B138-behavsci-15-01678] Wilkinson D. A., Johnson K., Garton H. J., Muraszko K. M., Maher C. O. (2017). Trends in surgical treatment of Chiari malformation type I in the United States. Journal of Neurosurgery: Pediatrics.

[B139-behavsci-15-01678] Wilson R. S., Krueger K. R., Gu L. P., Bienias J. L., De Leon C. F. M., Evans D. A. (2005). Neuroticism, extraversion, and mortality in a defined population of older persons. Psychosomatic Medicine.

[B140-behavsci-15-01678] Yang J., Bauer B. A., Wahner-Roedler D. L., Chon T. Y., Xiao L. (2020). The modified WHO analgesic ladder: Is it appropriate for chronic non-cancer pain?. Journal of Pain Research.

[B141-behavsci-15-01678] Yarbrough C. K., Greenberg J. K., Smyth M. D., Leonard J. R., Park T. S., Limbrick D. D. (2014). External validation of the Chicago Chiari Outcome Scale clinical article. Journal of Neurosurgery-Pediatrics.

